# Chabazite-supported ZnO and CeO_2_ nanoparticles with structural stability and enhanced antibacterial action

**DOI:** 10.1371/journal.pone.0344459

**Published:** 2026-03-13

**Authors:** José C. González-Crisostomo, Rigoberto López-Juárez, Ethiel Zavala-Flores, José Luis Villegas-Chávez, Ofelia Candolfi-Arballo, Víctor Alfredo Reyes Villegas, Jesús Isaías De León Ramírez, Vitalii Petranovskii

**Affiliations:** 1 Facultad de Ciencias Químicas e Ingeniería, Universidad Autónoma de Baja California, Tijuana, Baja California, México; 2 Instituto de Investigaciones en Materiales-Unidad Morelia, Universidad Nacional Autónoma de México, Morelia, México; 3 Facultad De Ciencias De La Salud, Universidad Autónoma De Baja California, Tijuana, Baja California, México; 4 Centro de Nanociencias y Nanotecnología, Universidad Nacional Autonoma de México, Ensenada, Baja California, México; National Chung Cheng University, Taiwan & Australian Center for Sustainable Development Research and Innovation (ACSDRI), AUSTRALIA

## Abstract

Chabazite (CHA) was synthesized by the hydrothermal fusion method, and zinc oxide/cerium oxide nanoparticles (ZnO/CeO_2_-NPs) supported on CHA were subsequently synthesized by the sol-gel method. The incorporation of the ZnO/CeO_2_-NPs (up to 25 wt.%) on structural, morphological and optical properties has been studied, and the applications of the materials in antibacterial studies have also been evaluated. XRD analysis confirmed the preservation of the zeolitic structure with progressive reduction of relative crystallinity. FTIR results showed that the CHA framework bands were preserved, although with evidence of surface interaction with the metallic phases. SEM and TEM images revealed a progressive coverage of the support with well-dispersed ZnO/CeO_2_ spherical NPs with average particle sizes between 8 and 9 nm, and EDX confirmed the homogeneous incorporation of ZnO/CeO_2_-NPs. UV-vis showed a shift of the absorption edge towards the visible range, attributed to Ce doping and modification of the electronic structure. BET analysis indicated an initial decrease in surface area due to pore blocking, followed by a recovery in samples with higher ZnO/CeO_2_-NPs loading, associated with a surface redistribution of the ZnO/CeO_2_-NPs and increased textural accessibility. Antimicrobial assays against *Pseudomonas aeruginosa (P. aeruginosa)*, *Enterococcus faecalis (E. faecalis)* and *Staphylococcus aureus (S. aureus)* showed concentration-dependent inhibition, being the gram-negative strains more sensitive. The synergistic antimicrobial effect was related to adsorption, generation of reactive oxidative species (ROS) and release of Zn^2+^. These multifunctional materials are promising for biomedical applications.

## Introduction

Chabazite-type zeolite (abbreviated as CHA) [1] has shown significant antibacterial activity in a number of studies [2–4]. This material, found in volcanic sediments, and similar to its synthetic counterparts, has the ability to adsorb and retain metal cations and molecules of organic compounds, which gives it antimicrobial properties [5]. The antibacterial activity of CHA has been associated with its capacity to host and release metal cations in a controlled manner, which may interfere with bacterial structure and cellular functions [6,7]. In addition, its high porosity and surface area favor interactions with a wide range or microorganisms, supporting its use as a functional support in antibacterial systems rather than as a standalone biocidal agent [8].

The natural and synthetic zeolites, including CHA-type structures have been widely explored in environmental [9], pharmaceutical [10], and biomedical-related applications due to their chemical stability, low cytotoxicity [11], and biocompatibility when used as inert supports rather than as active pharmaceutical agents [12].

The microporous crystalline structure of CHA belongs to the *R3m* space group [13], composed of two secondary Composite Building Subunits, D6R and CHA [14,15]. These subunits are linked together to form a three-dimensional lattice that forms nanometer-scale cavities and channels [16,17]. According to IZA, the available volume of CHA is 17.27% of the total windows in its channels along the a, b and c axes, which allow the diffusion of spherical particles with a diameter of 3.72 Å, and the maximum diameter of a sphere that can be incorporated into a cavity is 7.37 Å [18]. This structural arrangement is fundamental for its ability to absorb and accommodate molecules or nanoparticles (NPs), such as for example zinc oxide (ZnO) in its four cationic sites [19], enhancing, through confinement, the stability and catalytic activity of the NPs [20,21]. The CHA cavities provide stable support and a high surface area, promoting uniform dispersion and preventing agglomeration of ZnO NPs [22]. In addition, active zinc species can also be formed on the outer surface of zeolite crystals. ZnO NPs exhibit antibacterial, antitussive, photocatalytic and non-irritant and non-allergenic properties, as well as low toxicity [23]. Due to their high biocidal toxicity and strong photo-reducing action, they are considered an interesting type of useful materials for a wide range of applications, including organic agriculture, medicine, composites, closed environments, sunscreens, brake bands [24], the textile industry, electronic equipment, and automobiles [25].

Doping ZnO with cerium ions (Ce^3+^) has been shown to be an effective strategy to improve its antibacterial properties [26–28]. Cerium (Ce), a rare earth element, can alter the electronic structure and morphology of ZnO NPs, which increases their ability to generate reactive oxygen species (ROS), such as hydroxyl radicals and hydrogen peroxide [29,30]. These ROS are highly effective in damaging cell membranes and intracellular components of bacteria, which contributes to their destruction [23,31–33]. In addition, doping with Ce^3+^ ions improves the photocatalytic stability of ZnO, allowing it to maintain its antibacterial activity even under UV light exposure conditions [34]. In contrast, cerium oxide (CeO_2_) is a well-known n-type semiconductor [35], so it exhibits unique properties when doped with ZnO [36]. Its high ionic conductivity could be beneficial due to its exceptional ability to remove oxygen from the crystal lattice or surface [37], this process is known as oxygen storage capacity. This leads to the creation of oxygen vacancies [38], resulting in the formation of many active sites for chemical reactions to take place [37,39–41].

Beyond their antibacterial activity, ZnO and CeO_2_-based nanoparticles have also been reported to exhibit broader biological effects, including antioxidant, antifungal, and cytotoxic activities, depending on their size, surface chemistry, and oxidation status [42,43]. In particular, the redox cycle between Ce^3+^/Ce^4+^ in CeO_2_ has been associated with both ROS removal and ROS generation behavior, which underlies the biological responses mediated by oxidative and antioxidant stress that have been reported [44]. ZnO nanoparticles have also shown antifungal activity and concentration-dependent cytotoxic effects, mainly attributed to the generation of ROS and the release of metal ions [45,46]. These biological responses are strongly influenced by the scattering of the nanoparticles, surface interactions, and the nature of the supporting matrix.

ZnO and CeO_2_ nanoparticles (ZnO/CeO_2_-NPs) can be incorporated onto zeolites using methods such as wet impregnation, hydrothermal synthesis [30] or sol-gel method. The sol-gel method enables the integration of NPs into the zeolitic network, ensuring homogeneous dispersion and interaction with the zeolite’s active sites. This approach enhances the stability and functional properties of the materials, thereby improving their antimicrobial activity [27,47,48].

In this research, a combined synthesis was used to obtain a structurally stable zeolite support and, at the same time, achieve controlled dispersion of ZnO/CeO_2_-NPs. CHA was synthesized using the hydrothermal fusion method, which promotes high crystallinity, well-defined microporosity, and high ion exchange capacity. Subsequently, ZnO/CeO_2_-NPs were synthesized in situ and deposited on the CHA surface using the sol-gel method, selected for its ability to control the nucleation and growth on NPs, while promoting homogeneous dispersion and strong interfacial interaction with the zeolite structure. The objective of this study was to conduct a comprehensive investigation of this synthesis route and to evaluate the antimicrobial performance of the resulting ZnO/CeO_2_-NPs/CHA materials against *P. aeruginosa*, *E. faecalis* and *S. aureus*. In particular, this work addresses the lack of systematic studies correlating NPs loading, morphology, and dispersion with antibacterial efficiency in ZnO/CeO_2_/zeolite-based systems, highlighting the role of CHA as an effective multifunctional support.

## Experimental

### Materials

Sodium aluminate (NaAlO_2_, 53 wt% of Al_2_O_3_, 42.5 wt% of Na_2_O), sodium silicate pentahydrate (Na_2_SiO_3_·5H_2_O, 95%), potassium hydroxide (KOH, 90%), zinc nitrate hexahydrate (Zn(NO_3_)_2_·6H_2_O, 98%), Cerium nitrate hexahydrate (Ce(NO_3_)_3_·6H_2_O, 97%), ammonium hydroxide (NH_4_OH, 28% NH_3_ in H_2_O) were supplied by Aldrich Chemical.

### Synthesis of chabazite

**Fig 1:**
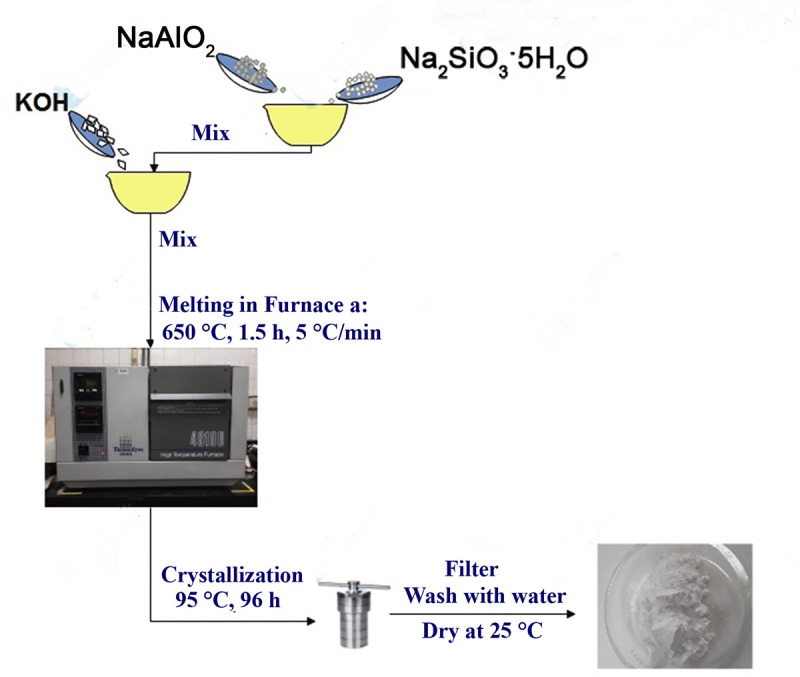
Diagram of the stages of synthesis of initial CHA.

In this research, CHA-type zeolite was synthesized by hydrothermal melting method. A certain amount of Na_2_SiO_3_-5H_2_O was mixed with NaAlO_2_ at a Si/Al molar ratio equal to 2. KOH was then added to the resulting mixture at a KOH/mixture mass ratio equal to 2.5. The mixture was melted in a furnace (Thermolyne 46100) at 650 °C for 1.5 hours. The resulting product was cooled to room temperature and dissolved in deionized water at a mass ratio of water/product of 4:1. The product was transferred to an autoclave for hydrothermal crystallization at 95 °C for 4 days (Fig 1). Finally, the precipitate was filtered, washed, and dried to obtain the initial CHA, which was subsequently used for ZnO/CeO_2_-NPs deposition experiments [6,49,50].

### Process of depositing ZnO and CeO_2_ NPs on CHA

ZnO/CeO_2_-NPs supported on CHA were synthesized by the sol-gel method according to the Zn_0.95_Ce_0.03_O general formula. The total loading of Zn_0.95_Ce_0.03_O on CHA support was systematically varied between 1, 5, 10, 15, 20, and 15 wt%, while maintaining the same Zn:Ce ratio in all samples. The corresponding amount of Zn(NO_3_)_2_·6H_2_O was dissolved in 20 mL of deionized water. Then, the corresponding amount of Ce(NO_3_)_3_·6H_2_O was dissolved in 5 mL of deionized water and added to the Zn(NO_3_)_2_·6H_2_O solution. After homogenizing the mixed solution, the pH was adjusted to 5 using NH_4_OH 0.1M. An appropriate amount of CHA was then added to the mixture, and the mixture was heated to 65 °C for several hours until the complete gel formation. The gel was dried at 100 °C for 24 hours to obtain a xerogel, which was placed in an oven (Thermolyne-46100) and calcined for two hours at 450 °C, at a heating rate of 1 °C/min in an air atmosphere [51]. The amounts of reagents used are summarized in Table 1.

**Table 1:** Amounts of chemical reagents used to synthesize a series of samples with different concentrations of ZnO/CeO_2_-NPs on CHA.

Samples	Weight of reagents (grams)		
	CHA	Zn(NO_3_)_2_·6H_2_O	Ce(NO_3_)_3_·6H_2_O
**ZC-01**	2.97	0.07	0.03
**ZC-05**	2.85	0.34	0.17
**ZC-10**	2.70	0.68	0.35
**ZC-15**	2.55	1.02	0.52
**ZC-20**	2.40	1.36	0.70
**ZC-25**	2.25	1.70	0.87

### Characterization and measurements

The phase composition of the synthesized samples was characterized by X-ray diffraction (XRD) using a Philips X-Pert MPD diffractometer. Diffraction patterns were recorded with Cu K_α_ radiation (λ = 1.54056 Å), employing a step size of 0.020° and a step time of 1.2 s per data point, over a 2θ range of 5° to 50°, allowing accurate identification of the crystalline phase, this data is presented as support information (S1 Data). The crystallinity of the samples presented in Table 2 was calculated by measuring the peak intensities at 2θ ≈ 9.05, 12.85, 16.96, 22.37, 30.54, 34.48, and 39.26° based on the following equation [52]:

**Table 2:** Crystallite size and relative crystallinity of the original chabazite and ZnO/CeO_2_-NPs synthesized chabazites different concentration.

Sample	Phase	Crystallite Size (nm)	Relative Crystallinity (%)
**CHA**	CHA	36.7	100
**ZC01**	CHA	–	36.76
	ZnO	–	
	CeO_2_	–	
**ZC05**	CHA	–	38.68
	ZnO	–	
	CeO_2_	–	
**ZC10**	CHA	19.3	47.12
	ZnO	51.3	
	CeO_2_	36.1	
**ZC15**	CHA	24.5	51.61
	ZnO	50.6	
	CeO_2_	27.1	
**ZC20**	CHA	18.6	49.78
	ZnO	49.1	
	CeO_2_	27.1	
**ZC25**	CHA	38.5	41.77
	ZnO	48.4	
	CeO_2_	23.0	


$$Crystallinity(\%)=\frac{\sum (mainpeaksof\;samples)}{\sum (mainpeaksofpure\;CHA)}\;X\;\text{1}\text{0}\text{0}$$ (1)

The morphology and structure of the synthesized samples were analyzed using a JEOL JSM-IT300 scanning electron microscope (SEM) and a JEOL JEM-ARM200F transmission electron microscope (TEM). Fourier transform IR spectra were recorded in the wavenumber range from 4000 to 400 cm^-1^ with a Thermo Fisher FTIR OMNIC 9 infrared spectrometer to identify functional groups, this data is presented as support information (S2 Data). Textural properties, including specific surface area (S_a_), micropore and mesopore surface area (S_micro_, S_meso_), pore volume (V_p_), micropore and mesopore volume (V_micro_, V_meso_) and average pore diameter (D_p_), were determined by adsorption/desorption of N_2_ at −196 °C using a Micromeritics Tristar II analyzer after degassing the samples at 300 °C under vacuum by 3 h [53,54]. The Langmuir, t-plot, Barret, Joyner & Halenda (BJH), Density Functional Theory (DFT) methods were applied for data analysis. Optical properties were examined by UV-Vis diffuse reflectance spectroscopy (DRS) in the 200–800 nm range with a Cary 5000 spectrometer, this data is presented as support information (S3 Data).

### Antibacterial study

The antibacterial properties of the obtained samples were analyzed by means of the macrodilution technique [55], using cultures of three bacterial species of medical-dental importance, all of them registered in the American Type Culture Collection (ATCC), namely: a Gram-negative *P. aeruginosa* (ATCC 27853), and both Gram-positive *E. faecalis* (ATCC 29212) and *S. aureus* (ATCC 25923). Primary bacterial cultures were grown on Trypticase Soy Agar (TBS, Beckton Dickinson), which was prepared according to the manufacturer’s instructions, the bacteria were inoculated and left to grow at 36 ± 1 °C for 24 hours. Two to three colony-forming units were taken to bring the bacterial solution to the 0.5 McFarland standard. Bacterial growth inhibition assays were performed in Muller Hinton liquid culture (Beckton Dickinson), from which 3 mL was placed in sterile glass tubes, adding 4 μL each of bacterial solution and the material to be evaluated.

The solutions were formed with each of the synthesized materials by dissolving 100 mg in 100 mL of sterile water; from this solution, four volumes were selected: 500 μL, 1000 μL, 1500 μL and 2000 μL, which were added to the tubes with the bacterial solution and liquid culture medium. After 24 hours of incubation under constant agitation at 36 ± 1 °C, absorbance at 650 nm was measured using Thermospectronic Genesys 5 equipment. All analyses were performed in triplicate. Bacterial cultures without material exposure were used as negative controls (bacteria-only control). For all the used strains, the antibiotics used to measure the desired effect (control +) were those provided in the ATCC® kit. (Red line). All antibacterial experiments were performed in triplicate. Statistical analysis was carried out using one-way analysis of variance (ANOVA) to evaluate the effect of concentration and material composition on bacterial inhibition. Differences were considered statistically significant at p < 0.05. Post hoc comparisons were performed when applicable to identify significant differences among groups.

## Results and discussion

**Fig 2:**
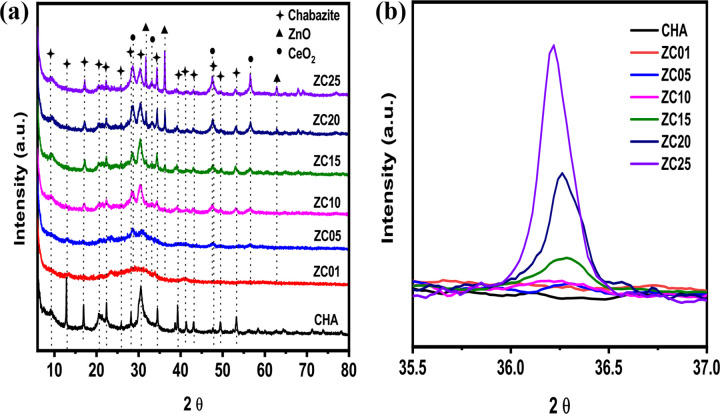
X-ray diffraction patterns. (a) XRD patterns for synthesized initial chabazite, and a series of chabazites with ZnO/CeO2-NPs. (b) magnified XRD patterns for all samples showing peak (101) for ZnO.

Fig 2a shows the X-ray diffraction patterns (XRD) of the synthesized CHA and ZnO/CeO_2_-NPs supported on CHA samples. The pure CHA sample shows a diffraction pattern characteristic of its crystalline phase, with well-defined peaks at 2θ ≈ 9.24°, 12.88°, 16.90°, 20.67°, 21.34°, 22.34°, 25.82°, 28.26°, 30.73°, 34.51°, 39.32°, 41.30°, 43.23°, 49° and 52° with corresponding interplanar distances (h k l) of 9.41 (1 0 0), 6.93 (−1 1 0), 5.06 (1 1 1 1), 4.34 (−2 1 0), 4.08 (2 1 0), 4.00 (−2 1 1 1), 3.46 (−2 2 0), 3.14 (3 0 0), 2.91 (3 1 0), 2.62 (−3 2 1), 2. 27 (−4 1 1), 2.17 (−4 2 0), 2.10 (−2 4 1) and 1.86 Å (−3 3 3), showing that the product consists of K-type CHA crystals, with the presence of cage structures specific to the CHA-type structure [19, 49, 56]. The ZC01 and ZC05 samples showed a decrease in the peak intensity without significant angular shift, indicating no alterations in the cell parameters or in the basic topology of CHA, with only a gradual loss of crystallinity (Table 2). This process was confirmed by determining the relative crystallinity of the samples using Equation 1. This decrease in crystallinity is a consequence of structural disorder caused by the incorporation of Zn and Ce species or possibly due to ion exchange without the collapse of the zeolitic structure, which is in agreement with previous studies [57]. Furthermore the possible dealumination and destruction of the CHA crystal structure suggests the appearance of a broad peak between 15° and 30°, centered at 2θ ≈ 22.9°, which is a typical characteristic of amorphous silica [58,59], but such a peak is absent in the ZC01 and ZC05 samples.

The presence of ZnO is evidenced in the sample ZC05 with additional peaks at 2θ ≈ 31.8°, 34.4°, 36.3°, 47.5° and 56.6°, corresponding to planes (100), (002), (101), (102) and (110) of hexagonal wurtzite-type structure (JCPDS data card no. 36–1451) [60,61]. The peaks reach their maximum intensity in sample ZC25, indicating further crystallization and accumulation of ZnO on the CHA surface. Also, in samples ZC10, ZC15, ZC20 and ZC25, characteristic CeO_2_ peaks located at 2θ ≈ 28.6°, 47.5° and 56.3° were detected, corresponding to planes (111), (220) and (311) of the cubic fluorite-type structure (JCPDS data card no. 34–0394) [62]. were detected. The appearance of these peaks confirms the presence of CeO_2_ NPs on the CHA surface, being more evident in ZC20 and ZC25 samples.

From the XRD diffractograms, the crystallite size was estimated using the Scherrer equation, τ = 0.9λ/βcosθ, where λ is the wavelength of X-ray radiation, β is the full width at half-maximum (FWHM) in radians, and θ is the diffraction angle of the peak with the highest intensity [63]. The following planes were used for the analyzed samples: (111) of CHA at 2θ ≈ 16.90°, (101) of ZnO at 2θ ≈ 36.3°, and (311) of CeO_2_ at 2θ ≈ 56.3°. The calculated crystallite size values for CHA samples with ZnO/CeO_2_-NPs are summarized in Table 2. It was observed that as the concentration of Zn and Ce increases in the samples studied, the crystallite size of ZnO decreases. This effect can be attributed to the difference in the ionic radii of Ce^3+^ (1.01 Å) and Ce^4+^ (0.97 Å), which are slightly larger than those of Zn^2+^ (0.74 Å) [64]. Fig 2b shows a magnification of the diffraction peak (101) of ZnO at 2θ ≈ 36.28°. In this Fig it is observed a shift towards smaller angles with increasing Zn and Ce concentration. According to the JCPDS crystallographic chart (No. 36–1451) for ZnO, the reported lattice parameter “*a*” is 3.250 nm, and “*c*” is 5.207 nm [65]. The lattice parameter “*a*” calculated from the (100) peak of ZnO is 3.250 nm for samples ZC15 and ZC20, and 3.254 nm for the ZC25 material. The lattice parameter “*c*” calculated from peak (002) was 5.216, 5.208 and 5.214 nm for samples ZC15, ZC20 and ZC25, respectively. This slight variation indicates the expansion of the ZnO crystal lattice, possibly attributed to internal stresses or structural distortions caused by interaction with Ce^3+^ [66]. Although the presence of peaks attributable to CeO_2_ indicates that Ce forms a separate crystalline phase, the observed displacement of the (101) ZnO peak suggests that some part of Ce may interact with the ZnO lattice. This interaction could be due to surface doping, partial substitution, or structural defects generated by the proximity between phases [67]. Doping of ZnO with Ce indicates local stresses due to the difference in ionic radii, which may lead to the expansion of the cell parameters [47,64].

**Fig 3:**
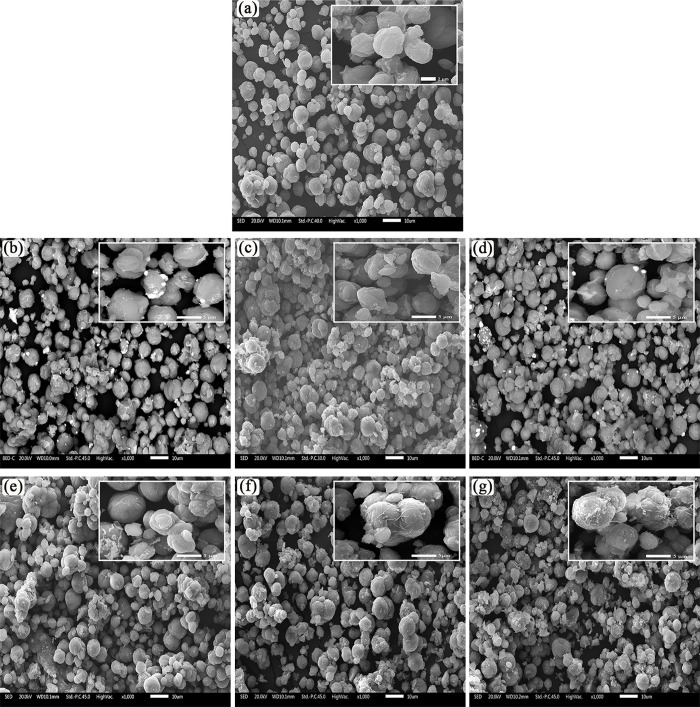
SEM images of synthesized native chabazite and samples with ZnO/CeO_2_-NPs. **(a)** CHA, **(b)** ZC01, **(c)** ZC05, **(d)** ZC10, **(e)** ZC15, **(f)** ZC20 and **(g)** ZC25.

The surface morphology of the synthesized materials is shown in Fig 3. Microstructural modifications induced by the progressive incorporation of ZnO/CeO_2_-NPs are clearly visible on the CHA particles, revealing a systematic evolution of NPs dispersion and aggregation as a function of loading. The micrograph corresponding to a pure CHA sample (Fig 3a) shows the characteristic morphology of well-defined crystals with angular contours, and a relatively clean surface, typical of zeolitic structures with high crystallinity [68]. In the samples ZC01 and ZC05 (Figs 3b and 3c), small and irregular crystals agglomerations are visible, indicating the initial nucleation of ZnO/CeO_2_-NPs on the available surface active sites of CHA [69]. In the ZC10 and ZC15 samples (see Figs 3d and 3e), a significant increase in surface coverage density is observed, with ZnO/CeO_2_-NPs forming discrete quasi-spherical aggregates.. This morphology indicate controlled growth and coalescence of crystalline domains [70]. At higher loadings in samples ZC20 and ZC25 (Figs 3f and 3g), where the zeolite surface appears abundantly covered by a dense layer of ZnO/CeO_2_-NPs, indicating significant aggregation and partial loss of the original CHA morphology. This behavior suggests an oversaturation of the active anchoring sites, which promotes secondary nucleation on the pre-existing NPs rather than on the support, resulting in more pronounced aggregation. No structural collapse or formation of large particles is observed in all samples, suggesting that the synthesis method allows good control over the dispersion and size of the active phases.

The progressive deposition and accumulation of ZnO/CeO_2_-NPs as aggregates on the surface of CHA crystals can be also confirmed by analyzing the elemental composition of the samples by X-ray energy dispersive spectroscopy (EDX) (Table 3), which supports the results obtained by XRD and SEM analyses. The pure CHA sample exhibits a typical profile for the composition of CHA-type zeolite composed mainly of oxygen (O, 60.96 at.%), silicon (Si, 16.49 at.%), aluminum (Al, 12.67 at.%) and potassium (K, 9.88 at.%) with a Si/Al ratio of 1.30, which is consistent with a CHA structure with high cation exchange capacity [1,49,50,71]. The incorporation of ZnO/CeO_2_-NPs to the ZC01 sample reveals the presence of Zn (2.34 at.%) and Ce (0.95 at.%), indicating successful and uniform deposition at low concentration. The oxygen content increases significantly compared to pure CHA, which is attributed to the presence of ZnO and CeO_2_ NPs on the CHA surface. With increasing concentration of ZnO/CeO_2_-NPs, there is a progressive increase in the proportion of Zn and Ce in the elemental composition. In particular, the Ce content steadily increases to 6.46 at.% for ZC25, while the Zn content also increases, although with a slight decrease for ZC25, which may indicate a redistribution between surface and volume, or a limitation of the load-bearing capacity for Zn at elevated concentrations. At the same time, a systematic decrease in Si, Al and K content is observed, indicating partial coverage of the CHA surface by ZnO/CeO_2_-NPs, and possibly also a partial ion exchange of K^+^ ions for Zn^2+^ ions during the synthesis process, which is consistent with the data obtained from SEM images. Moreover, this decrease is also consistent with the relative attenuation of CHA peaks in the XRD patterns for ZC20 and ZC25 samples, in which the peaks of ZnO and CeO_2_ nanocrystals are markedly intensified. It is worth mentioning that according to the EDX results ZnO/CeO_2_-NPs are preferentially deposited on the surface of CHA crystals, forming heterogeneous layers. No evidence of framework incorporation or substitution of Si or Al atoms was observed, indicating that ZnO/CeO_2_-NPs are predominantly deposited on the external surface of CHA.

**Table 3:** Average elemental composition of synthesized chabazite and samples containing ZnO/CeO_2_-NPs.

Atomic, %	CHA	ZC01	ZC05	ZC10	ZC15	ZC20	ZC25
O	60.96	65.67	67.39	66.48	67.26	66.66	66.42
Si	16.49	11.51	10.21	10.27	9.35	9.26	8.67
Al	12.67	9.68	9.77	9.45	8.44	8.42	8.10
K	9.88	9.84	6.42	6.80	5.54	6.26	6.45
Zn	--	2.34	2.04	2.10	4.43	4.39	3.90
Ce	--	0.95	4.16	4.89	4.99	5.01	6.46
**Si/Al**	**1.30**	**1.19**	**1.05**	**1.09**	**1.11**	**1.10**	**1.07**

**Fig 4:**
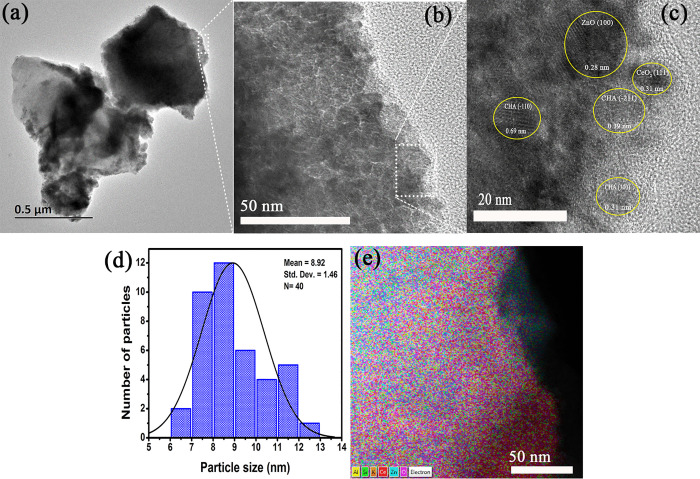
Transmission electron microscopy of the ZC05 sample. (a – c) TEM images, (d) histogram of particle size distribution and (e) EDS mapping for ZC05 sample.

Fig 4 shows TEM images of ZnO/CeO_2_-NPs supported on CHA for sample ZC05, selected as representative of low-to-intermediate NPs loading. The low magnification micrograph (Fig 4a), shows the characteristic morphology of the CHA support with irregularly shaped agglomerated particles, while darker contrast regions correspond to ZnO/CeO_2_-NPs deposited on the CHA surface. This observation confirms the surface-localized deposition inferred from SEM and XRD analyses. Higher magnification image (Fig 4b) show well-defined domains homogeneously distributed on the CHA surface, indicating effective recoating at this load level. The high-resolution image (Fig 4c), clearly shows crystalline planes with interplanar distances of 0.28 nm and 0.31 nm, corresponding to the (100) ZnO [72] and (111) CeO_2_ [73] planes, respectively. Additionally, planes with interplanar distances of 0.31, 0.39 and 0.69 nm, corresponding to the (300), (−211) and (−110) planes of CHA, respectively [74], were observed. The identification of the phases by HRTEM method agrees with the crystalline phases identified by XRD. Fig 4d shows the statistical analysis of particle size, the average particle size distribution was 8.92 ± 1.46 nm. The small size and narrow distribution of the observed ZnO/CeO_2_-NPs explains why ZnO/CeO_2_-NPs are not clearly defined and confined in the SEM images (Fig 3). Elemental mapping (Fig 4e) shows a homogeneous distribution of Zn and Ce on the CHA surface, confirming an efficient distribution without phase segregation.

**Fig 5:**
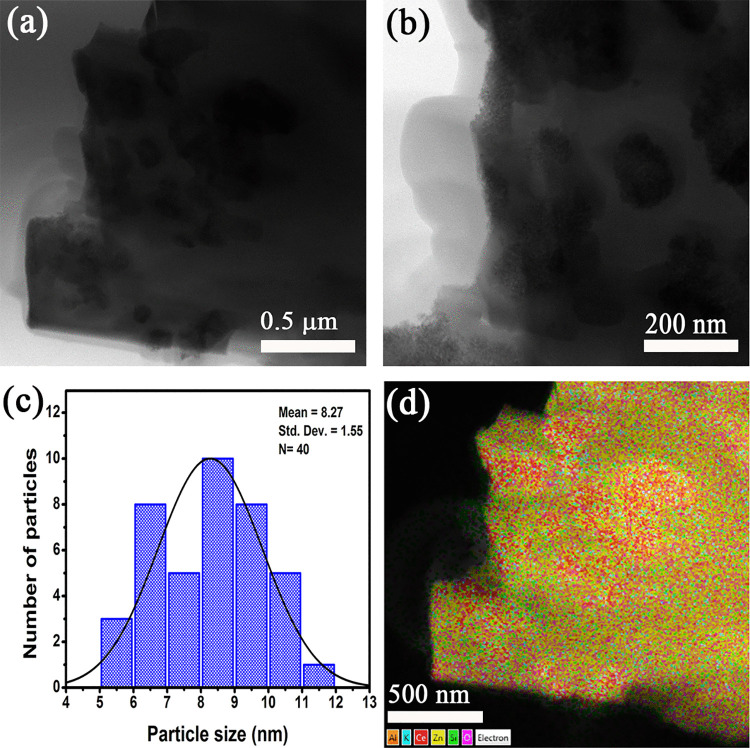
Transmission electron microscopy of the ZC25 sample. (a and b) TEM images, (c) histogram of particle size distribution and (d) EDS mapping for ZC20 sample.

Fig 5 shows TEM images, size histogram, particle size distribution and elemental mapping of the ZC25 sample. Fig 5a shows a structure consisting of dark particles with high electron density distributed on CHA (light-colored support). The contrast intensity and the number of visibly detectable NPs is significantly higher compared to the sample with lower ZnO/CeO_2_-NPs content, such as ZC05 (Fig 4), indicating the dense and extensive deposition of ZnO/CeO_2_-NPs on CHA. Fig 5b shows that the ZnO/CeO_2_-NPs have spherical or quasi-spherical morphologies, forming dense agglomerates that tend to continuously cover the CHA surface. Fig 5c shows a histogram of the particle size distribution, based on the measurement of 40 NPs, with an average size of 8.27 ± 1.55 nm. Although this average value is slightly smaller than that of the ZC05 sample, the range of dispersion is similar. However, the observed degree of agglomeration indicates that at relatively high concentrations of ZnO/CeO_2_-NPs, as is the case of sample ZC25 with a concentration of 25 wt%, nucleation on pre-existing NPs is favored, leading to heterogeneous growth on CHA, with supersaturation of active sites leading to agglomerate formation. The elemental mapping shown in Fig 5d indicates a homogeneous distribution of Zn and Ce, confirming the existence of ZnO/CeO_2_-NPs on the CHA surface.

**Fig 6:**
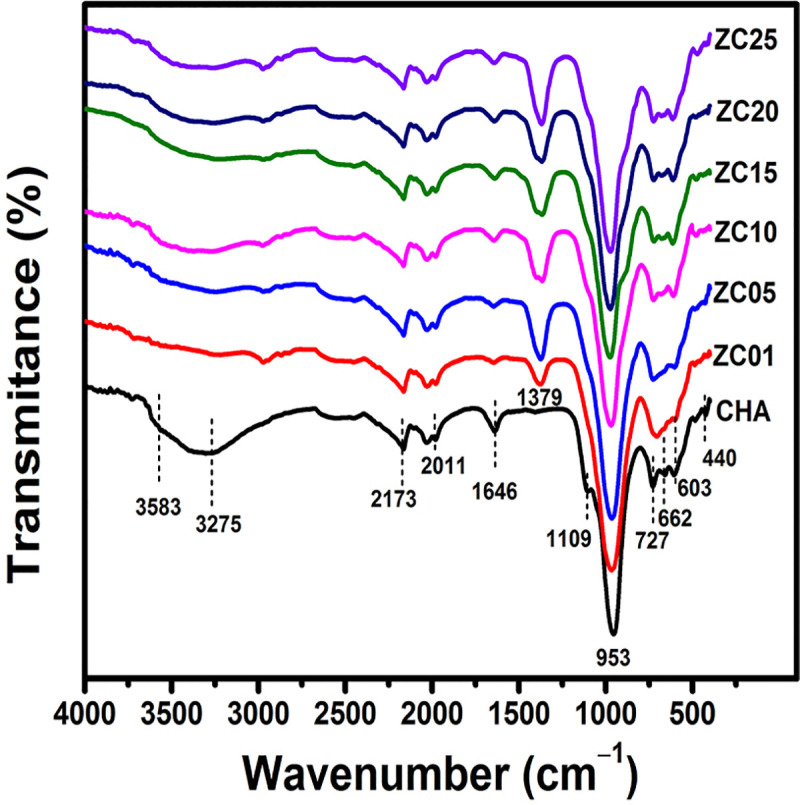
FT-IR spectra of synthesized chabazite and ZnO/CeO_2_-NPs supported chabazite materials.

For the pure CHA and the ZnO/CeO_2_-NPs covered samples, the FT-IR spectra in the 4000−400 cm^-1^ region were investigated, shown in Fig 6. Three important sub-regions can be distinguished in all the spectra. The vibrations between 1600 and 3700 cm^-1^ can be attributed to the presence of water in CHA matrix. The band at 3583 cm^-1^ is attributed to the interaction between OH groups of water and K^+^ cations [75]. The bands at 1646 and 3275 cm^-1^ in the synthesized CHA are attributed to stretching vibrations and bending vibration of O-H, respectively, and the possible presence of structural or adsorbed water. The persistence of these signals in all samples indicates that surface hydration is maintained, although a slight reduction in their intensity is observed with increasing concentration of ZnO/CeO_2_-NPs, which is probably due to the occupation of surface sites by the NPs and thus limiting water adsorption.

Besides water, zeolites also adsorb carbon dioxide. The absorption band at 2173 cm^-1^ is due to the bond vibrations of the CO_2_ molecule upon its absorption from air. The peak at 1379 cm^-1^ corresponds to the C = O bond associated with the CO_2_ molecule [51].

The bands associated with Si-O-Si and Si-O-Al vibrations and Si-O or Al-O bridging bonds in the basic constituents of the CHA crystal structure are in the wavenumber region of 400–1200 cm^-1^ [76]. The bands at 1109 and 953 cm^-1^ are related to symmetric and asymmetric stretching vibrations of Si-O-Si and Si-O-Al, or stretching vibration of SiO_4_ units in the CHA structure. The band at 723 cm^-1^ is attributed to the symmetric stretching vibrations of Si-O-Si, while the signal at 603 cm^-1^ is related to the coupling vibrations of the TO_4_ tetrahedra (T = Si, Al) in the CHA structure [50, 77–79]. Moreover, the signal at 662 cm^-1^ is related to the external and internal vibrations of the six-membered double ring [80,78]. The absence of the band at ~450 cm^-1^, associated with the bending vibrations of Si-O-Si indicates the absence of amorphous silica in the samples [81]. These results agree with the XRD patterns (Fig 2a), which do not exhibit the broad diffuse halo centered around 2θ ≈ 22.9°, a distinctive feature of amorphous silica. The preservation of the characteristic Si-O-Si and Si-O-Al vibrational bands, together with the absence of new metal-oxygen framework bands, further supports that the interaction between ZnO/CeO_2_-NPs and CHA is mainly due to surface deposition rather than incorporation into the zeolite framework.

Table 4 summarizes the results of surface area and textural properties of the synthesized samples. The pure initial CHA presented a moderately low surface area (4.45 m^2^/g) and pore volume (0.0027 cm^3^/g). These values are lower than the typically reported for natural CHA (521–846 m^2^/g and 0.255–0.47 cm^3^/g, respectively [82]. This low surface area is consistent with synthetic or ion exchange CHA, where the pores are blocked or there is an incomplete development of the microporous network during synthesis tends to result in smaller accessible surfaces. Ridha et al. [83] reported that surface area analysis using N_2_ at 77K may considerably underestimate the actual porosity of K-exchanged CHA. This is due to the narrow pore openings, which limit N_2_ diffusion, resulting in values as low as 17.82 m^2^/g. However, the intrinsic porosity measured with CO_2_ adsorption is much higher. Similarly, interzeolitic transformation pathways have shown to yield CHA with relatively low surface areas [84], reinforcing the idea that the low surface area observed here is likely intrinsic to the synthesis method and not a collapse of the crystal structure. After deposition of ZnO/CeO_2_-NPs, the ZC01 and ZC05 samples showed a decrease in surface area (2.18 and 2.08 m^2^/g, respectively), and pore volume by 0.00220 and 0.00208 cm^3^/g, respectively. This decrease in surface area and pore volume may be due to the partial pore obstruction due to the ZnO/CeO_2_-NPs deposition on the CHA surface, which is also manifested by the decrease in crystallinity observed in the XRD. However, an increase in surface area is observed in the subsequent samples, which reaches 4.67 m^2^/g in ZC10, 3.70 m^2^/g in ZC15, 6.63 m^2^/g in ZC20 and 11.50 m^2^/g in ZC25. The same trend is observed in the pore volume, which increases from 0.00282 cm^3^/g in ZC10 to 0.00644 cm^3^/g in ZC25. This slight increase in surface area is not attributable to the reopening of zeolitic micropores, rather, it is due to the formation of secondary mesopores associated with aggregation and gaps between ZnO/CeO_2_-NPs, as evidenced by SEM (Fig 3) and TEM (Fig 5) micrographs.

**Table 4:** Textural properties of synthesized chabazite and of the chabazite with ZnO/CeO_2_-NPs.

Samples	*S*_*a*_^a)^	*S*_*micro*_^b)^	*S*_*meso*_^c)^	*V*_*p*_^d)^	*V*_*micro*_^b)^	*V*_*meso*_^c)^	*V*_*p*_^e)^	*D*_*p*_^b)^	*D*_*p*_^e)^	*D*_*p*_^f)^
	(m^2^/g)			(cm^3^/g)				nm		
CHA	4.45	4.16	0.29	0.0027	0.0014	0.0013	0.0016	1.31	1.45	1.49
ZC01	2.18	2.18	0.00	0.0022	0.0012	0.0010	0.0012	2.21	2.23	1.50
ZC05	2.08	2.08	0.00	0.0021	0.0010	0.0011	0.0021	2.00	3.94	1.51
ZC10	4.67	4.59	0.08	0.0028	0.0015	0.0013	0.0016	1.32	1.39	1.09
ZC15	3.70	3.49	0.21	0.0025	0.0012	0.0013	0.0013	1.33	1.43	1.50
ZC20	6.63	6.42	0.21	0.0040	0.0022	0.0018	0.0025	1.30	1.48	1.50
ZC25	11.50	10.57	0.94	0.0064	0.0033	0.0031	0.0044	1.13	1.53	1.48

*S*_*a*_= surface area, *S*_*micro*_= micropore surface area (*t*-plot), *S*_*meso*_= mesopores surface area (*S*_*a*_-*S*_*micro*_), *V*_*p*_= pore volume, *V*_*micro*_= micropore volume (*t-*plot), *V*_*meso*_= mesopore volume (*S*_*a*_-*S*_*micro*_), *D*_*p*_= average pore diameter, Measured by ^a)^ Langmuir, ^b)^ *t*-plot, ^c)^ *S*_*a*_-*S*_*micro*_, ^d)^ DFT, ^e)^ D-A= Dubinin-Astakov, ^f)^ HK=Horvath-Kawazoe Method

**Fig 7:**
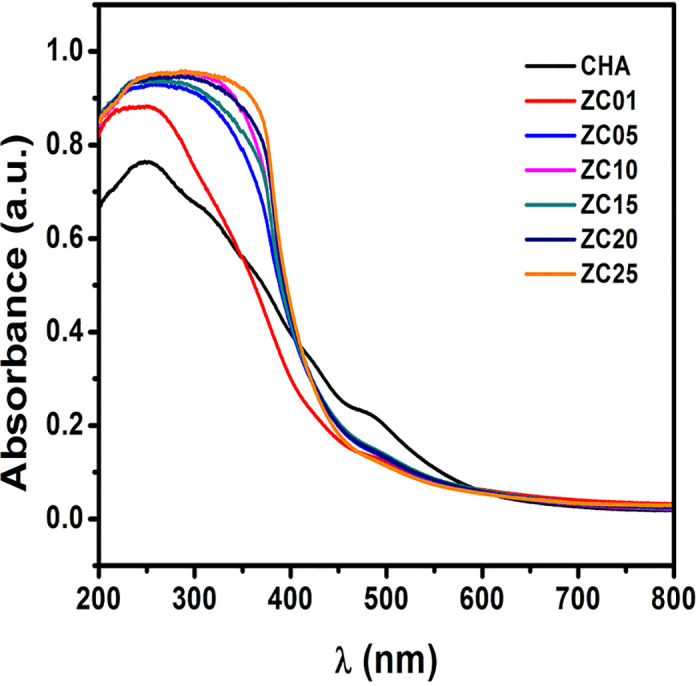
UV-vis DRS spectra of synthesized chabazite and ZnO/CeO_2_-NPs deposited chabazite materials.

The UV-Vis spectra of synthesized CHA and CHA with deposited ZnO/CeO_2_-NPs are shown in Fig 7. CHA exhibits optical absorption limited to the far UV. Upon addition of ZnO, a characteristic absorption band in the near-UV (~ 330−390 nm) appears associated with the ZnO bandgap transition (E_g_ = 3.2–3.3 eV). With the incorporation of Ce, a redshift of the absorption edge towards longer wavelengths, i.e., towards lower energy, and an overall increase in absorbance is observed. In addition, a Ce^3+^ absorption band (4f → 5d transition) centered near 309 nm was identified, which becomes more prominent in samples with higher Ce content [27].

**Fig 8:**
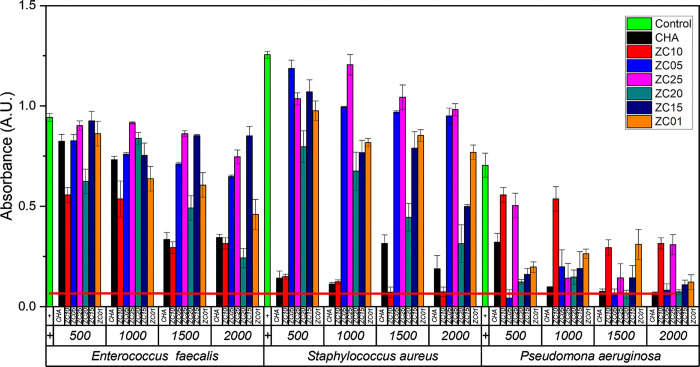
Absorbance of *E. faecalis*, *S. aureus*, and *P. aeruginosa* exposed to CHA-type zeolites with varying contents of ZnO/CeO_2_-NPs. Four concentrations (500–2000 µg/mL) were tested and compared with the unmodified sample (CHA).

The activity against the Gram-negative *P. aeruginosa* follows the same trend for all the samples (Fig 8). Yet, a different sensibility is observed in the presence of ZC10 and ZC25 materials, the bacteria proliferation increases, with a monotonic change positively correlating with concentration. The same trend was detected in ZC15 and ZC20 samples with a stronger growth inhibition. This monotonic change suggests that mechanism of inhibition is associated with only one phenomenon. This behavior indicates the predominance of a single inhibition mechanism, which, based on the lack of enhanced inhibition compared to CHA, can be mainly attributed to bacterial adsorption onto the zeolite surface. Given the monotonic inhibition effect when Zn and Ce were added, and the lack of increase in inhibition compared to CHA, the inhibition mechanism should be attributed to adsorption. As for the observed non-monotonic changes in some samples, the differences in inhibition can be attributed to a modified mass transfer upon adsorption of bacteria onto the zeolite surface, primarily on the introduced ZnO/CeO_2_-NPs [85]. In these cases, secondary mechanisms related to nanoparticle–bacteria interactions may compete with adsorption. In the case of *P. aeruginosa*, a Gram-negative bacterium, a higher negative potential is detected, promoting greater interaction with CeO_2_ NPs [26,86]. These interactions are influenced by the surface charge and morphological features of the nanoparticles, including their dispersion and aggregation state on the CHA surface.

As for the ZC01 and ZC05 samples, growth inhibition follows a non-linear concentration dependence, with fluctuations being less efficient at concentrations of 1500 and 1000, respectively. Nevertheless, the ZC05 material can be distinguished by higher growth inhibition at concentrations of 500, 1500 and 2000. Hence, the mechanism of antimicrobial action, according to the growth profile, follows a non-monotonic change in inhibition. This non-monotonic behavior suggests the coexistence of more than one antibacterial mechanism. Its complex behavior is due to an active mechanism consisting of more than one phenomenon, such as surface adsorption, and those related to the role of introduced NPs. For example, competition between adsorption and other mechanisms, perhaps the generation of ROS by CeO_2_ NPs [26], or the oligodynamic action of the potential antivirulence agent of ZnO [87]. The incorporation of ZnO and CeO_2_ can also generate ROS by coupling the introduced band gap of the nanoparticles (Fig 7) when supported on the CHA framework leading to a more reactive surface due to the increased surface defects (Fig 3 and Fig 4). Showing the potential synergistic recombination of antimicrobial mechanisms between the individual components of ZnO/CeO_2_-NPs [88].

The inhibition varies depending on the type of bacteria, which is explained by the electrical potential generated by the different structures of their cell walls. In the case of *P. aeruginosa*, a Gram-negative bacterium, a higher negative potential is detected, favoring a stronger interaction with CeO_2_ NPs (Table 5) [26,86,89]. In contrast to *P. aeruginosa, E. faecalis* and *S. aureus*, both Gram-positive, tend to adhere to more positively charged particles. On this basis, the ZC10, ZC15, ZC20 and ZC25 samples should have similar growth inhibition. Nevertheless, given the lower inhibition detected in these samples, the mechanism must be related to the generation of ROS or the potential antivirulence action of ZnO [87].

**Table 5:** Summarizes the antibacterial performance of the CHA-based materials investigated in this work in comparison with previously reported zeolite and metal oxide-based antibacterial system.

Material	Bacterial strains	Concentration range (µg/mL)	Main antibacterial mechanism	Key findings	Ref.
CHA	*E. faecalis*, *S. aureus*, *P. aeruginosa*	500–2000	Bacterial adsorption	Moderate inhibition and a concentration-dependent	This work
ZnO/CeO_2_ NPs	*E. faecalis*, *S. aureus*, *P. aeruginosa*	500–2000	Bacterial adsorption, ROS generation, and metal–cell interaction	Enhanced inhibition for *P. aeruginosa,* with non-monotonic trends in some ratios	This work
ZnO NPs	*E. coli, E. faecalis*	100–3000	ROS generation, Zn^2 +^ release	Stronger effect on Gram-negative bacteria	[85]
ZnO NPs	Gram-negative and gram-positive	1.5- 25 wt%	Bacterial adsorption and oligodynamic effect	Concentration-dependent inhibition	[90]
CeO_2_ NPs	*S. mutans*	7-8%	ROS generation and membrane interaction.	Higher inhibition for biofilm bacteria	[26,86]
CeO_2_ NPs	*E. coli S. typhimurium S. aureus B. cereus*	50	Redox imbalance and oxidative stress.	Uniform nanoparticle distribution improves inhibition	[89]
ZnO	*P. aeruginosa*	20–100	ROS generation and Zn^2 +^ ion release	Size and concentration-dependent activity	[88,91]

**Fig 9:**
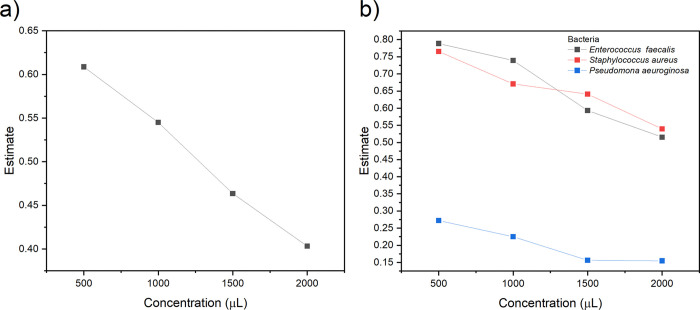
(a) Evaluation of the antibacterial effect of CHA samples with ZnO/CeO_2_-NPs applied at different concentrations. (b) Comparative ANOVA evaluations for *E. faecalis*, *S. aureus*, and *P. aeruginosa* showing concentration-dependent decrease in viability.

Likewise, previous studies have also shown that CeO_2_ NPs can inhibit bacterial growth through various mechanisms, one of the most significant being their interaction with the bacterial cell wall. This inhibition process is determined by the interaction that CeO_2_ NPs establish with bacteria. Although the interaction with *E. coli* has been studied previously, it has been observed that in the case of *P. aeruginosa*, the distribution of NPs over the bacteria is more uniform, which may explain the differences in the inhibition between Gram-positive and Gram-negative bacteria, which are influenced by the adsorption of NPs on their surfaces [26,86,89]. ANOVA analysis showed that there was a clear concentration dependence of inhibition, regardless of the microorganism (Fig 9a). However, when inhibition was tested across the microorganisms examined, this inhibition was more strongly observed in *P. aeruginosa* than in *E. faecalis* and *S. aureus* (Fig 9b). This suggests that the inhibition mechanism in *P. aeruginosa* (gram-) is less sensitive compared with that in gram+ bacteria (*E. faecalis* and *S. aureus*). This difference may be associated with variations in ROS generation and lifetime, which are influenced by the optical properties of the ZnO/CeO_2_-modified materials. The antibacterial evaluation against Gram-negative bacteria was limited to *P. aeruginosa*. Future studies will include additional Gram-negative strains to further validate the observed inhibition mechanisms.

**Fig 10:**
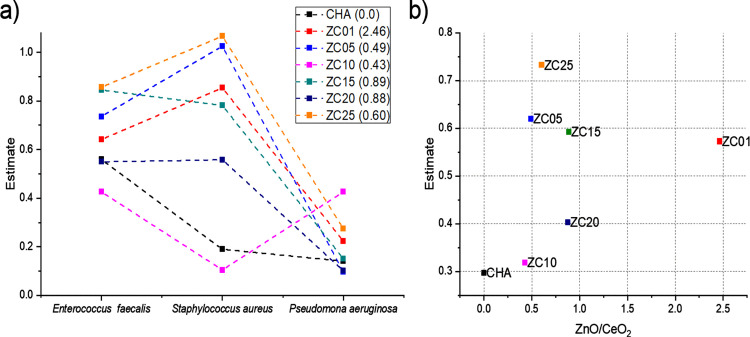
(a) Effect of the ZnO/CeO_2_ ratio on the antibacterial effect and B) evaluations against for *E. faecalis*, *S. aureus*, and *P. aeruginosa* of CHA samples with ZnO/CeO_2_-NPs applied at different concentrations.

From this statistical analysis, the inhibition mechanisms for *E. faecalis* and *S. aureus* may be similar, given that they are both Gram+ and are affected by the charge and structural composition of their membranes (Fig 10a). Since the materials surface repel them, the mechanism of inhibition would be mostly related to the diffusion of ROS. This effect is modulated by nanoparticle morphology, surface defects, and agglomeration, as observed in the SEM analysis. The ZC10 sample exhibits a trend similar to CHA, suggesting that both share comparable adsorption mechanisms. However, the Zn and Ce species present in this sample should be different from those in other samples, given the observed changes when analyzing different samples. For *P. aeruginosa*, synergism between adsorption and nanoparticle addition was detected, while the inhibition effect was slightly less in this ZC10 sample. This indicates that adsorption or higher concentrations of Ce and Zn are needed to achieve a significant effect. Nonetheless, the similarities in samples ZC01 and ZC20 against *E. faecalis* and *P. aeruginosa* suggest that these can be sensitive to specific metal species, regardless of the ZnO/CeO_2_ ratio (Fig 10b).

No clear trend is observed when analyzing the ZnO/CeO_2_ ratio, however, the ZC10 sample showed potential against *E. faecalis* and *S. aureus*, while ZC20 was effective against *E. faecalis* and *P. aeruginosa*. The complex behavior results from the intricate antibacterial mechanisms associated with each metal oxide. For ZnO, antibacterial action is attributed to multiple mechanisms, including direct interaction with the bacterial cell wall, generation of Zn^2+^ ions, and production of ROS [92]. These processes are strongly influenced by the optical band gap and surface defect density of ZnO nanoparticles. Zn^2+^ ions enhance antimicrobial activity by interfering with the regulation of some virulence factors, such as CZC pump (Cobalt/Zinc/Cadmium), while ROS induce oxidative stress that contributes to cell damage [88,90,91]. On the other hand, CeO_2_ NPs can also deactivate essential enzymes that protect bacteria from oxidative stress by increasing the level of ROS inside cells, causing oxidative stress, and leading to bacterial death [26].

Interestingly, the bacteria do not appear to be affected by ZC25 material; if any effect is observed, it becomes saturated at concentrations above 1500 μL, likely due to metal agglomeration. This behavior is consistent with morphological observations, where nanoparticle aggregation reduces the effective surface area available for bacteria–particle interactions. Statistical analysis indicates that higher concentrations are associated with direct metal interaction, while at lower concentrations, adsorption is the predominant mechanism. The nanostructure of ZnO, including features such as Schottky barriers and surface phonon activity, also plays a key role in mediating the interplay between physical and chemical mechanisms, thereby enhancing antimicrobial efficacy. The activity of ZnO is influenced by several factors, including the size and shape of the NPs. The ZnO (111) facet exhibits the highest antimicrobial activity. The effectiveness of NPs depends on both size and concentration: smaller particles with larger surface area exhibit greater antibacterial ability, while higher concentration of ZnO was observed to increase bacterial mortality. Additionally, surface defects and orientation of NPs contribute to their biocidal activity [88].

It is evident from the present study that the interaction of bacteria with CHA results in inhibition of their growth. However, treatments of CHA with some concentrations of ZnO/CeO_2_-NPs can have a synergistic effect that enhances the inhibition of Gram-negative bacteria. The proposed mechanism is complex, and is mainly due to the adsorption of bacteria onto the zeolite surface, which is then enhanced by adsorption attributed to ZnO and CeO_2_. The incorporation of ZnO and CeO_2_ modifies the surface charge, optical response, and morphology of CHA, which collectively influence electrostatic interactions and ROS-related processes. Probably, after the introduction of ZnO and CeO_2_, the surface charge of the zeolite particles changes, which leads to a change in the electrostatic interaction, and adsorption of the particles on the bacterial membranes. This change may reduce antimicrobial activity against Gram-positive bacteria, since the electrostatic interaction between bacteria and the surface of the material is a critical factor in bacterial inhibition. The antibacterial behavior discussed above is strongly supported by the morphological characteristics revealed by SEM and TEM analyses. The nanoscale size of the ZnO/CeO_2_ particles, their dispersion on CHA surface, and the progressive aggregation observed at higher loadings directly influence bacterial adsorption, mass transfer, and the activation of additional mechanisms such as ROS generation and metal ion interaction.

## Conclusions

Initial chabazite (CHA) in K^+^-ion-exchange form was successfully synthesized by the hydrothermal fusion method and subsequently modified with ZnO/CeO_2_-NPs deposited on its surface using the sol-gel method. Structural analyses confirmed that the CHA crystalline framework was preserved after NPs incorporation, although a gradual attenuation of the CHA diffraction peaks was observed at higher loads due to surface coverage by ZnO/CeO_2_-NPs, with no evidence of amorphous phase formation. SEM and TEM analyses demonstrated the effective dispersion of ZnO/CeO_2_-NPs on the CHA surface, with average particle sizes in the range of 8–9 nm and increasing surface coverage as NPs loading increased. EDX mapping further confirmed the homogeneous distribution of Zn and Ce on the CHA surface, supporting the formation of heterogeneous ZnO/CeO_2_-NPs layers rather than bulk doping of the CHA framework. Textural analysis revealed an initial decrease in surface area associated with pore blocking effects, followed by partial recovery at higher NPs contents, which can be attributed to the redistribution of accessible surface area between CHA and the supported ZnO/CeO_2_-NPs, consistent with the observed morphological evolution. Optical characterization by UV-Vis diffuse reflectance spectroscopy indicated notable changes in absorption behavior upon NPs incorporation, including a red shift associated with Ce^3+^ electronic states and interfacial interactions between ZnO, CeO_2_ and CHA. Antibacterial tests against *P. aeruginosa*, *E. faecalis* and *S. aureus* showed a clear concentration-dependent inhibitory effect, with Gram-negative bacteria showing higher susceptibility. Among the studied materials, sample ZC20 exhibited the most balanced antibacterial performance across all tested strains, suggesting an optimal compromise between NPs loading, dispersion, and surface accessibility. Finally, it should be noted that antibacterial activity was evaluated using inhibition assays, while minimum inhibitory and bactericidal concentrations were not determined, and ROS involvement was discussed based on literature reports rather than direct quantification. Cytotoxicity or biocompatibility assessments were also beyond the scope of the present work. These aspects will be addressed in future studies to further elucidate the antibacterial mechanisms and assess the suitability of ZnO/CeO_2_-NPs/CHA composites for biomedical applications. Overall, these results demonstrate that CHA serves as an effective support for the dispersion of ZnO/CeO_2_-NPs, preserving structural integrity while enhancing physicochemical and antibacterial properties, making these composites promising multifunctional materials for antimicrobial and related applications.

## Supporting information

S1 Data Raw data for X-ray diffraction. Data used for calculated parameters and calculations of the samples used.
(XLSX)

S2 Data Raw data for Fourier transform IR spectra. Data used from the samples used.
(XLSX)

S3 Data Raw data for UV-Vis diffuse reflectance spectroscopy. Data used from the samples used.
(XLSX)
